# The prevalence of metabolic syndrome up to 5 years post-partum in patients with a history of gestational diabetes mellitus

**DOI:** 10.1186/1753-6561-6-S4-P44

**Published:** 2012-07-09

**Authors:** C Crowe, E Noctor, LA Carmody, B Wickham, G Avalos, G Gaffney, P O’Shea, F Dunne

**Affiliations:** 1 Department of Medicine, University Hospital Galway and National University of Ireland, Galway, Ireland; 2 Department of Obstetrics and Gynaecology, University Hospital Galway, Ireland; 3 Department of Clinical Biochemistry, University Hospital Galway, Ireland

## Introduction

Metabolic syndrome (MetS) is associated with cardiovascular mortality and increased risk of type 2 diabetes.

## Methods

We examine the prevalence of MetS in a cohort of Caucasian women with previous gestational diabetes (GDM) (n=116), and those with normal glucose tolerance (NGT) during pregnancy (n=51). Fasting glucose alone (known DM/pre-diabetes post-partum patients) or 75g OGTT (other patients), lipid profile, insulin and c-peptide were performed. We calculated insulin resistance using the HOMA2-IR computer model.

## Results

**Table 1:** 

	Number with MetS (ATPIII criteria) (%)	Mean HOMA-2IR (+/- SD)	Number with HOMA-2IR>1.7 (%)
DM/pre-diabetes post GDM (n=33)	12 (36%)	1.21(+/-0.77)	18 (55%)
NGT post GDM (n= 83)	11 (13%)	1.93(+/-1.4)	18 (22%)
No history GDM (n=52)	3 (6%)	1.02(+/-0.85)	6 (13%)

## Conclusions

Metabolic syndrome and insulin resistance are significantly more prevalent in Caucasian patients with GDM progressing to post-partum DM/pre-diabetes than those who do not (p<0.01), suggesting a need to target lifestyle changes in the early post-partum period to help prevent progression to T2DM.

